# Analysis of Patterns of Bushmeat Consumption Reveals Extensive Exploitation of Protected Species in Eastern Madagascar

**DOI:** 10.1371/journal.pone.0027570

**Published:** 2011-12-14

**Authors:** Richard K. B. Jenkins, Aidan Keane, Andrinajoro R. Rakotoarivelo, Victor Rakotomboavonjy, Felicien H. Randrianandrianina, H. Julie Razafimanahaka, Sylvain R. Ralaiarimalala, Julia P. G. Jones

**Affiliations:** 1 School of the Environment, Natural Resources and Geography, Bangor University, Bangor, Gwynedd, United Kingdom; 2 Durrell Institute of Conservation and Ecology, University of Kent, Kent, United Kingdom; 3 Madagasikara Voakajy, Antananarivo, Madagascar; University of Jyväskylä, Finland

## Abstract

Understanding the patterns of wild meat consumption from tropical forests is important for designing approaches to address this major threat to biodiversity and mitigate potential pathways for transmission of emerging diseases. Bushmeat consumption has been particularly poorly studied in Madagascar, one of the world's hottest biodiversity hotspots. Studying bushmeat consumption is challenging as many species are protected and researchers must consider the incentives faced by informants. Using interviews with 1154 households in 12 communes in eastern Madagascar, as well as local monitoring data, we investigated the importance of socio-economic variables, taste preference and traditional taboos on consumption of 50 wild and domestic species. The majority of meals contain no animal protein. However, respondents consume a wide range of wild species and 95% of respondents have eaten at least one protected species (and nearly 45% have eaten more than 10). The rural/urban divide and wealth are important predictors of bushmeat consumption, but the magnitude and direction of the effect varies between species. Bushmeat species are not preferred and are considered inferior to fish and domestic animals. Taboos have provided protection to some species, particularly the Endangered Indri, but we present evidence that this taboo is rapidly eroding. By considering a variety of potential influences on consumption in a single study we have improved understanding of who is eating bushmeat and why. Evidence that bushmeat species are not generally preferred meats suggest that projects which increase the availability of domestic meat and fish may have success at reducing demand. We also suggest that enforcement of existing wildlife and firearm laws should be a priority, particularly in areas undergoing rapid social change. The issue of hunting as an important threat to biodiversity in Madagascar is only now being fully recognised. Urgent action is required to ensure that heavily hunted species are adequately protected.

## Introduction

Research spanning several decades has established that hunting of wildlife in tropical forests is a significant threat to biodiversity [1]–[3] and there is increasing concern about the risks it poses to public health through the transmission of zoonotic viruses [4]. Understanding the patterns of such bushmeat hunting is therefore important for designing appropriate management approaches to conserve threatened species [5], [6], and for mitigating possible transmission pathways of emerging diseases [7]. There have been a number of attempts to investigate predictors of bushmeat consumption. For example, wealth is an important predictor of consumption of animal protein in general but will be a stronger predictor of bushmeat consumption where wild meat is preferred over domestic meat [8]–[10]. Employment alternatives for hunters [11], access to guns or snares required for hunting [12] and variables such as livelihood activities and location [13] may also predict availability of bushmeat and therefore, to some extent, its consumption. The level of exploitation faced by particular species may be influenced by traditional taboos [14], [15] or, where laws are effectively communicated and enforced, by the degree of legal protection [16]. Despite the obvious potential for interactions between these various influences, there are few studies which consider the importance of socio-economic factors, preference, laws and traditional taboos as predictors of bushmeat consumption together.

Collecting reliable information on bushmeat consumption is difficult because many species are protected under national laws, meaning that informants may be unwilling to discuss their involvement to avoid incriminating themselves [17], [18]. Researchers wishing to build up a clear picture of patterns of consumption therefore need to explicitly consider incentives faced by their informants and attempt to triangulate their evidence from multiple sources wherever possible. Asking informants to recall recent events, such as meals they have eaten, is a commonly used method to collect information on individual behaviours and has the advantage of providing information from a specific time period, tying behaviour to an individual and, if recent events are used, reduces the risk of inaccuracies being introduced due to imperfect memory [19]. However, when the question is sensitive, recent recall questions may not be honestly answered. Focusing questions less specifically on a particular time period can reduce sensitivity but has obvious disadvantages; for example it is difficult to be certain whether patterns revealed are currently relevant. Questionnaire based surveys can be triangulated with direct observations of behaviour, helping to overcome that problem [20]. Few bushmeat studies have explicitly considered incentives based by informants and triangulation of data sources.

Madagascar is widely recognised as a global conservation priority [21]. Interest in hunting as an important threat facing the country's endemic fauna has lagged behind interest in bushmeat hunting in other tropical regions, but a growing number of reports suggest that hunting and consumption of wild animals may be more widespread than previously thought [22]–[26]. Despite evidence that hunting of some species is unsustainable and a serious threat to biodiversity [27], there have been no broad, systematic surveys of the extent and patterns of bushmeat consumption. We report the results from the first large scale study of the consumption of bushmeat in eastern Madagascar investigating patterns of consumption with respect to socio-economic variables, taste preference, and traditional taboos. We use field observations from locally based monitors to confirm some of our most important findings.

## Methods

### Study Area

The hunting of wild animals in Madagascar is governed by a clear legal framework which classifies species as strictly protected, protected, game or nuisance [28]. This study was carried out in towns and villages in two districts (Moramanga and Anosibe An'ala) in the Alaotra-Mangoro region of eastern Madagascar (Figure S1). Land use is a mixture of agricultural land, grassland, natural humid forest and exotic tree plantations. Access to the natural forest, and hunting and collecting of forest products is strictly controlled in three protected areas and around a nickel mine. Elsewhere in the region, significant areas of forest are being designated as new community-managed protected areas where access to forest resources is locally managed. The economy is agriculture-based and incomes are low (annual household expenditure in 2005 was between US$169 in rural areas and US$185 in urban areas, [29]).

As well as research permits from the national government, we obtained permission from the relevant local authorities and our researchers were accompanied in the field by a local guide appointed by village leaders. The fact that members of our team had worked in the area for a number of years and were generally well liked and trusted, helped to reassure informants that we were researchers and not associated with law enforcement agencies. We used a combination of approaches: recall questions about meals eaten in the last three days, questions about lifetime consumption, and direct recording by local monitors of bushmeat passing through a sub-set of villages.

### Ethical statement

This research has been approved by the ethics committee of the College of Natural Sciences, Bangor University, and also followed Madagasikara Voakajy's ethics policy. We were careful to ensure we obtained the informed consent of our research participant: the aims of the study and how data would be stored and used were explained to informants and that their participation was voluntary was clearly explained. Because of the low level of literacy in the area, we did not obtain written consent. To reduce the risk of harm to our informants, no identifying information was collected from informants and the location of study villages will be anonymised when the data is made available outside the core research team (e.g. for data archiving purposes). We do not identify the location of our sample villages in this paper.

### Household Interviews

Household interviews were conducted between January 2008 and October 2009 by five of the authors (AR, HJR, SR, VR, FHR), all Malagasy and familiar with culture, dialect and customs in the study area. Within each district we worked in rural communes with extensive humid forest and stratified our sample to ensure that households from the only two urban communes were adequately represented. In total, our sample contained data from 1,154 households in 12 different communes. We discarded 52 other interviews before analysis where the respondent was not deemed able to answer for the whole household.

A zig-zag route was taken through settlements and every third household was sampled [8]. If the head of the household, or whoever was present, didn't want to take part in an interview we moved onto the next household. Overall < 10% of households refused to participate in the study. We asked the respondents to recall what they had eaten in recent meals. A pilot study suggested that respondents' ability to recall declined substantially after the third day so questioning was limited to the previous three days. Next, we showed a series of 50 photographs of wild and domestic animals found in the region (Table S1) and asked whether respondents had, in their life time, ever eaten the species. When no consumption of the species was reported, we asked why not (whether they didn't recognise the species, it was not available, it was taboo *fady*, or they simply didn't want to eat it). The respondent was then invited to indicate the ten animal species that were most preferred, and to rank them in order of preference. Finally, we collected additional information about the socio-economic characteristics of the household. This included whether the family considered themselves to be long term resident (*tompontany*) or immigrants to the area (*mpiavy*); the principle source of household income (farming, commerce or salaried work); whether the household used wood alone for fuel or also used charcoal and gas and the number of rooms within the household (1, 2, or 3+). These last two were chosen as indicators of wealth [30] as experience of the area suggests that having more than one room, and using charcoal and/or gas is an indicator of relative wealth. Households in the two towns were classed as urban and all other households were classed as rural.

### Local monitoring

We supplemented our household interviews with observations collected directly by local monitors within a single commune in the Moramanga District. We employed 13 local monitors based in eight different *fokontany* administrative units to record wild animal carcasses, cooked meat or living individuals that were observed for sale, or consumed or transported through their village. In three localities, monitors operated simultaneously and we cross-checked data entries to ensure that there were no obvious double-counting (e.g. records of the same species on the same day). Monitors received 10,000 Ariary (US$5) per month and were recruited and trained by VR, a native of the region who had worked in the communities for over five years. In total we collected 135 months of data between March 2008 and June 2010. Monitors will differ in their access to information so data from different villages can't be directly compared as an index of bushmeat consumption. Similarly, monitors varied in what species they reliably recorded: with some recording any wild meat including fish, while other focused on mammals. However the data is useful to confirm which mammal species are being consumed in the study area and provides an estimate of minimum numbers killed.

### Statistical Analysis

We examined the relationships between patterns of consumption and households' socio-economic characteristics by fitting a series of statistical models to the data. Modelling was carried out in R 2.11.0 [31].

Six categorical variables were considered as potential predictors of consumption: whether the household was in an urban or rural area; whether the family considered themselves to be local or immigrants; the principle source of household income; whether the type of cooking fuel used and the number of rooms within the household. The timing of questionnaire surveys was determined by factors related to funding and the availability of human resources. We recognise that seasonality probably plays an important role determining patterns of bushmeat consumption in the study region but we omitted season as a predictor from the analysis because data from urban areas were only collected during a single season. Our variable indicating whether a household was urban or rural must therefore be interpreted in this light. Initial inspection of the data showed that the type of fuel used and livelihood activities of respondents was strongly associated with whether or not a household was located in an urban area, with rural households mostly occupied by farmers who used wood for fuel (Table S2). We therefore excluded the variables for livelihood activity and fuel type.

Our first set of models investigated which factors predict the proportion of respondents' meals which contained domestic or wild meat. Vector generalised linear models (VGLMs) with multinomial error families and logistic link functions were fitted to the three-day recall data using the vglm function from the VGAM package [32]. All combinations of the predictor variables were represented in the candidate set. In recall surveys, respondents' abilities to accurately recall their activities may not be consistent throughout the stated time period [33], [34]. However, initial exploratory analyses satisfied us that the reported patterns of consumption were not affected by the specific day within the three day period on which a meal was consumed. We therefore analyzed the data at the household level and each independent data point reflects three days consumption, with the response modelled as a three level, multinomial variable indicating how many of the household's meals over the preceding three days contained no animal protein, protein from domestic animals, or protein from wild-caught animals.

Next, a set of generalised linear mixed-models was fitted to the lifetime consumption data for strictly protected, protected or game species, using the lme4 package [35]. In this case the response was binary, indicating whether or not the respondent had ever consumed the species in question, so our models used binomial error families and logistic link functions. The same three predictor variables were considered for inclusion in the models, along with a further variable representing the species. To account for the grouping structure of the data, in which every individual responds to questions about the same set of species, we fitted individual respondent as a random intercept term. We also fitted interaction terms between species and the other fixed predictors. We considered a candidate set of seven models: a model containing all main effect and interaction terms, three models in which one interaction between species and the other predictors was removed, and three in which the corresponding main effect was also removed. The strength of the evidence for each model in both analyses was evaluated using Akaike's information criterion (AIC); [36].

To illustrate the findings from our models, we used them to produce average predictive comparisons of consumption under different scenarios (APCs; [37]). APCs are calculated by performing simulations from the fitted models in which the values of one or more focal variables are systematically varied, while all others are held constant. We incorporated uncertainty in parameter estimates into the predictions by simulating every scenario 1000 times, each time drawing parameter values at random from Normal distributions whose means and standard deviations equalled the means and standard errors of the fitted models' parameter estimates [37]. Finally, respondents' expressed preferences for species they had previously tasted were examined using a simple scoring system. A preference score was calculated for all species with sufficient data (arbitrarily defined as having been consumed by 10 or more respondents) as the proportion of respondents who placed it within their five most-preferred species to eat, resulting in a readily interpretable value between 0 (not preferred by any respondents who had eaten the species) and 1 (preferred by all respondents who had eaten it).

Basic descriptive statistics are provided from the data collected by local monitors. These data are summarised based on the protected status and taxonomy of the species observed as bushmeat. A more detailed analysis is presented for results pertaining to lemurs, including a breakdown of the number of each species observed in each locality during the study.

## Results

### Description of the sample

The 1,154 households with which we carried out interviews were split 11.4% urban and 88.6% rural. The majority of people in our rural sample classify themselves as farmers (more than 70% in all wealth categories), while more than 60% of urban people in all wealth categories say their livelihood is based in salaried work. There were slightly higher numbers of migrants in the urban than the rural samples (36.2% and 27.4% respectively).

### Three-day consumption recall

Of 3425 meals sampled in our dataset, the majority (74.5%) contained no animal protein, 11.8% contained protein from domestic animals and 13.7% contained protein from wild-caught animals. Of the 469 meals containing wild meat, the majority were fish and aquatic invertebrates, with only 9.6% from terrestrial wild animals (1.3% of all meals). The proportion of meals reported to contain meat from legally protected species (i.e., those categorised as strictly protected or protected) was very small (18 meals, or 0.5%).

**Figure 1:**
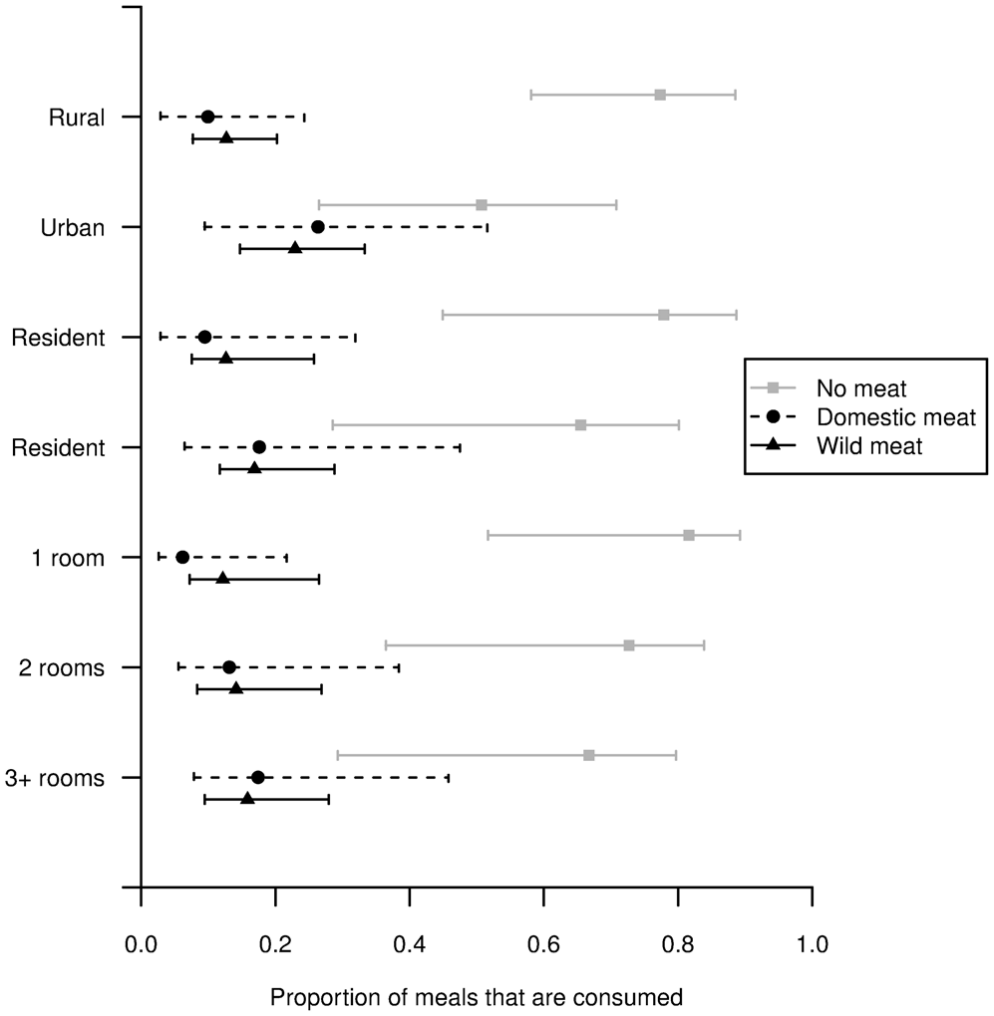
Average predictive comparisons generated from the best-fitting model for the three day recall data. Points mark the mean of the predictions, while the horizontal bars indicate the range into which the central 95% of predictions fall.

There was strong support for the model which included all three predictor variables (Tables S3 and Table S4). Predictions generated from the fitted model show the influence of the modelled predictors on the consumption of animal protein in Malagasy households (Figure 1). Urban households consume approximately twice as many meals containing meat as rural households on average (52.8% and 25.8% respectively), and migrants consume nearly twice as many meals containing meat than residents (41.9% and 29.4% respectively). Similarly, the proportion of meals containing meat is higher in households with a greater number of rooms, with households having three or more rooms consuming on average 60% more meals containing meat than single-room households (41.4% and 25.8% respectively). The size of the effect of each of the three variables was greater for domestic meat that for wild meat.

### Lifetime consumption

Despite the low proportion of meals containing meat from wild-caught animals, and the small percentage of meals reported to include meat from legally protected species, many individuals report having eaten protected species at some point in their lives. From the raw data, 95% admit to having eaten a protected or strictly protected species, and 44.5% have eaten 10 or more protected or strictly protected species. 96 percent have eaten game species.

**Figure 2:**
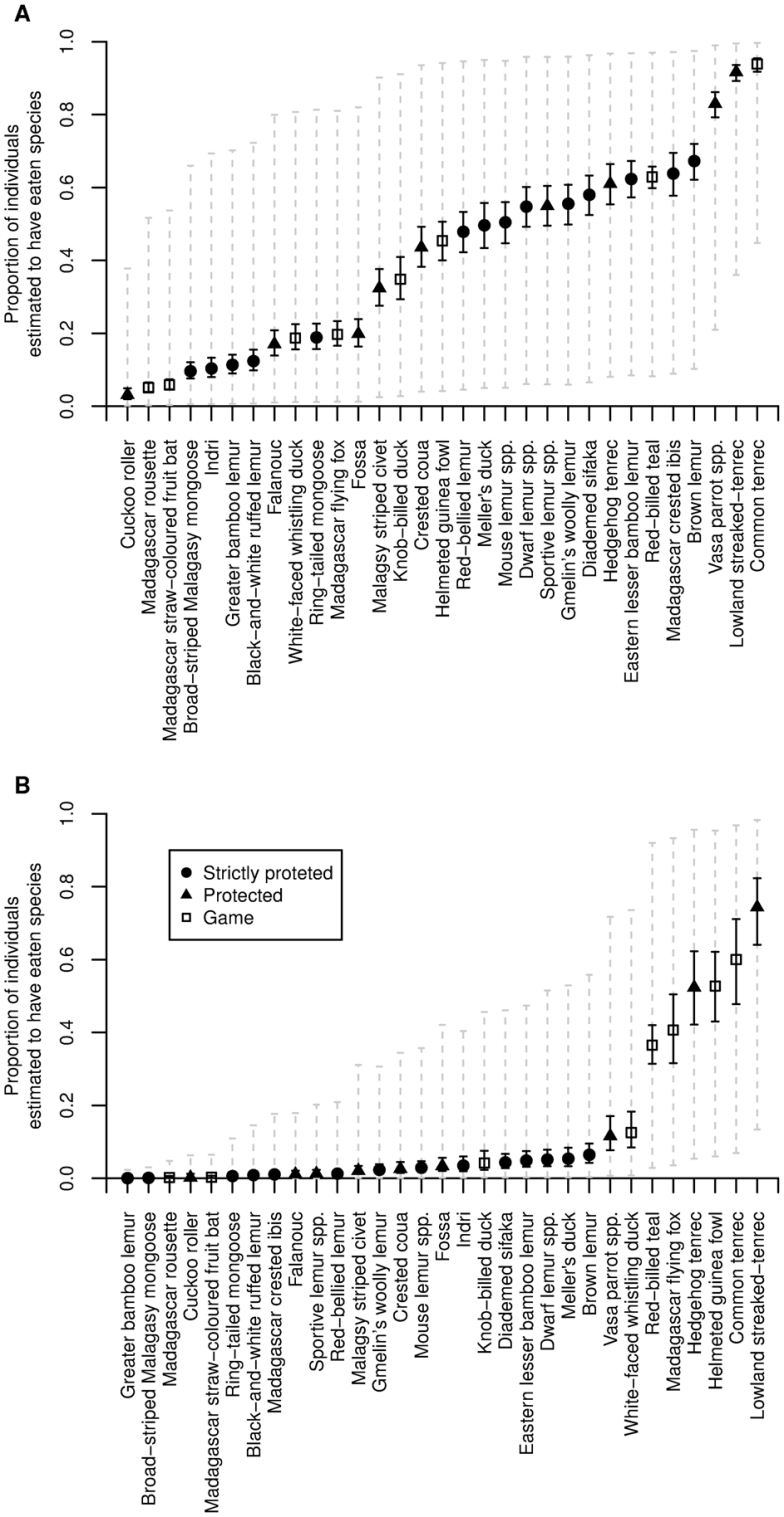
Estimated proportion of individuals who report that they have ever consumed a sample of 31 species classified as strictly protected, protected or game under Malagasy law. The two scenarios, illustrate the variability between species, and between types of household (A; rural living in single-roomed house, B; urban living in a house with 3 or more rooms). Points indicate the mean of predictions, solid vertical black lines indicate the variability in prediction attributable to parameter uncertainty, while grey dashed lines indicate the range of variability attributable to additional heterogeneity between respondents.

There was strong support for a single model including all of the main effects and interactions (Table S5 and Table S6). Predictions generated for two scenarios - one for individuals from poorer rural households, the second for richer urban households - show that the proportion of respondents who have ever eaten legally protected species varies greatly between species (Figure 2). Some, such as the lowland streaked-tenrec *Hemicentes semispinosus*, have been eaten by the majority of individuals while others, such as the cuckoo roller *Leptosomus discolor* have been eaten by very few individuals (Figure 2). The probability that an individual has eaten a species varies considerably according to their socio-economic characteristics with poor, rural people being much more likely to report having eaten protected species. Seven strictly protected, four protected and two game species (including several threatened lemurs) have been eaten by more than half of poorer rural households. By contrast, only two protected species and two game species have been eaten by more than half of richer urban households (Figure 2). The role of various socio-economic predictors on consumption varies between species. For example diademed sifaka *P. diadema* has been consumed by a much higher proportion of poorer, rural people (58% of whom have eaten this Endangered species) than richer, urban households (4%), while an urban household is twice as likely to have consumed Madagascar flying fox *Pteropus rufus* as a rural household (20% and 41% respectively, Figure 2).

**Figure 3:**
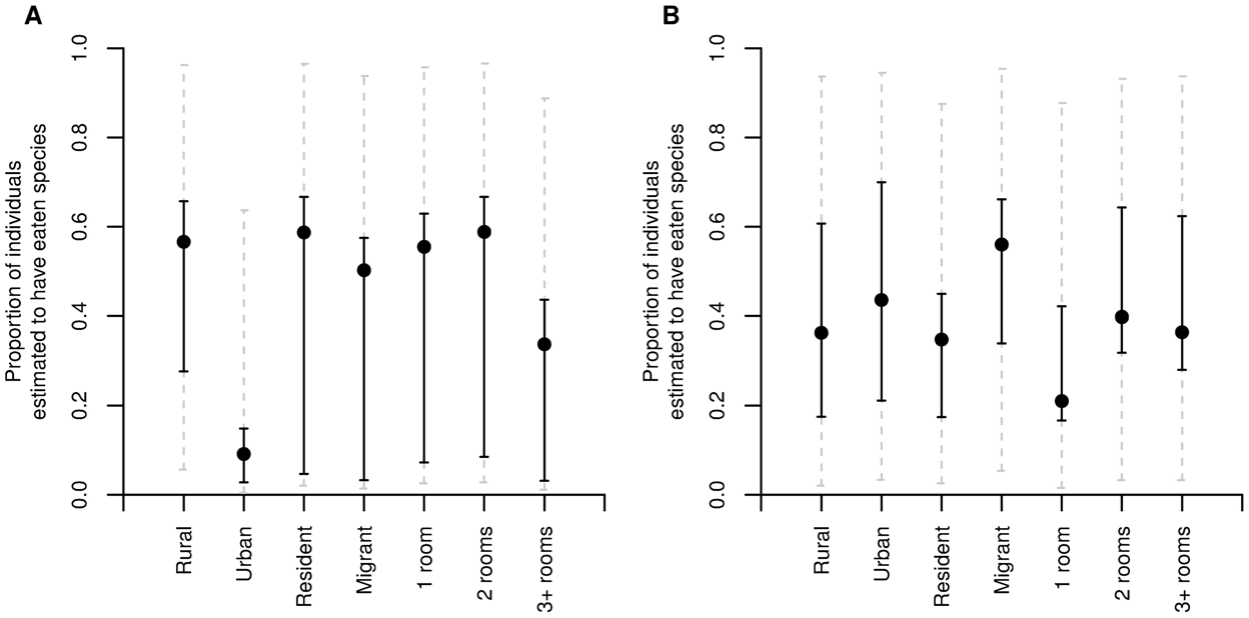
Average predictive comparisons for two species (A; diademed sifaka, B; Madagascar flying fox) illustrating the contrasting effects of modelled variables on the proportion of individuals predicted to have eaten the species. Points indicate the mean of the predictions and solid black lines show the range of variability attributable parameter uncertainty. Dashed grey lines indicate the variability attributable to additional heterogeneity between respondents.

Average predictive comparisons showing the effect of the three modelled predictors for these species are given in Figure 3. For the sifaka, the effect of coming from an urban household compared to a rural household is a very pronounced reduction in the probability that an individual has eaten the species, and a smaller reduction for individuals coming from households with three or more rooms (more wealthy households). For the Madagascar flying fox *P. rufus*, wealth is again an important predictor of consumption but in the opposite direction with wealthier households (more rooms) being more likely to report having consumed this species.

**Figure 4:**
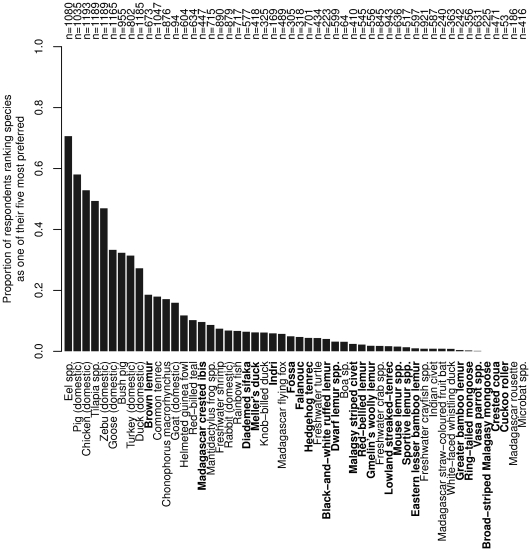
Ranking of species according to respondents' expressed preferences for meat. Bold species names indicate species that are strictly protected or protected under Malagasy law. n is the number of respondents who had eaten the each species.

The meats which were most frequently ranked amongst respondents' five most preferred were domestic animals such as pig, chicken, zebu, duck and goose, along with wild eel (*Anguilla* spp.), various tilapia (*Tilapia* spp.) and bush pig *Potamochoerus larvatus* (Figure 4). Protected species were generally not reported to be most preferred, although the brown lemur *Eulemur fulvus* was ranked in the top five most preferred species by 19% of respondents. A similar percentage ranked the common tenrec *Tenrec ecaudatus*, a game species, in their top five.

**Figure 5:**
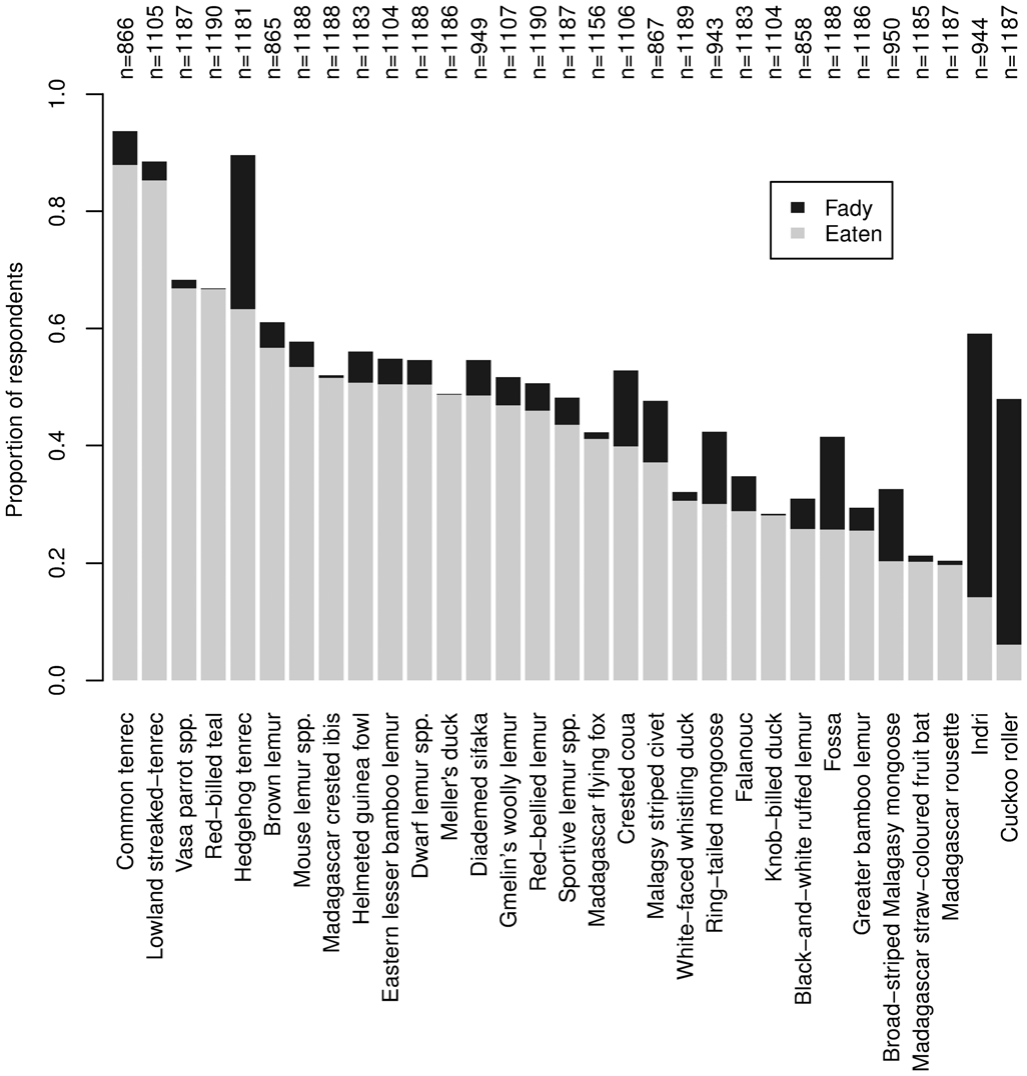
Proportion of respondents who reported that they were taboo (*fady*, black bars) for strictly protected or protected species, ordered by the proportion of respondents who have ever eaten the species in question (grey bars). The height of the black bars therefore represents the maximum difference in consumption that could be attributable to taboos for each species. n is the number of respondents who answered questions about each species.

There is considerable variation between species in the proportion of respondents who report that they do not eat them because of the existence of taboos (Figure 5). The proportion of individuals who were *taboo* was less than 7% for most legally protected species. Some, such at the hedgehog tenrec, fossa and crested coua, however, were taboo for more than 10% of respondents. The cuckoo roller and the Indri were reported as taboo by 42% and 45% of respondents, respectively (Figure 5).

### Local Monitoring

**Figure 6:**
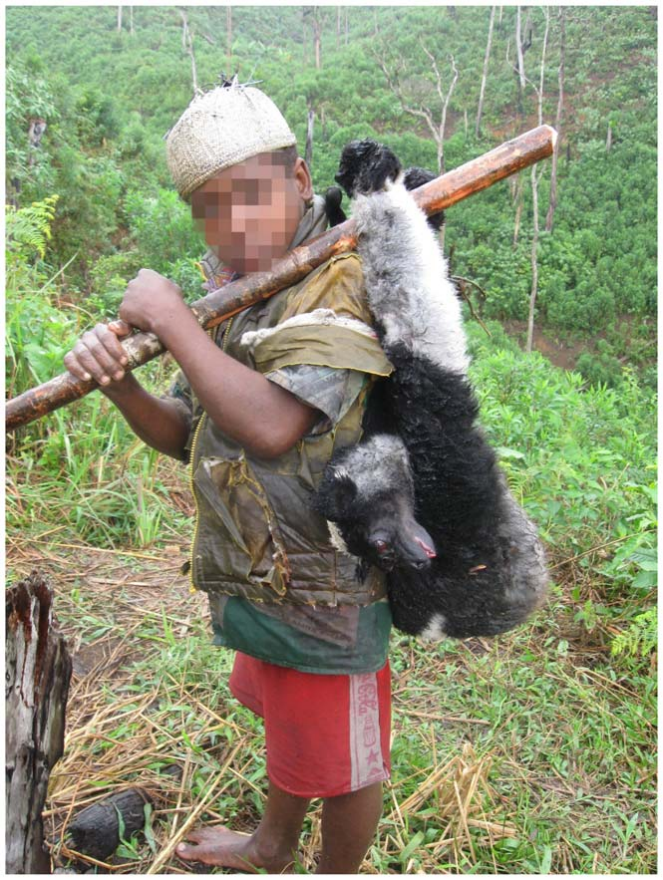
Boy carrying a recently killed Indri (the largest remaining lemur in Madagascar). Although this species has been protected to some extent by traditional taboos in our study area, social change appears to eroding this taboo resulting in an increase in hunting. His face is blurred to protect his identity © Madagasikara Voakajy.

A total of 489 mammal observations were noted in the logbooks including 246 records of strictly protected species (mostly lemurs). The 244 lemur records (representing 483 individuals) included at least nine species including nine individuals of the Critically Endangered black-and-white ruffed lemur *Varecia varecia* (see Table S7). The Endangered Indri (Figure 6) and diademed sifaka are the most frequently recorded lemurs with 121 and 233 individuals recorded. Most of these individuals were recorded by four monitors working at two sites but at least three individuals of both species were recorded by 10 and 12 of the 13 monitors respectively, giving us confidence that these species are being widely hunted in the area.

## Discussion

### Frequency and range of bushmeat consumption

In common with rural populations in many low-income countries [38], animal protein is rarely consumed in the study region. The animal protein that is consumed comes equally from domestic and wild sources. Although we came across few records of eating bushmeat in the three-day recall data, many respondents have consumed a wide variety of wild animal species listed as protected under Malagasy law. At least ten lemur species including the Critically Endangered Greater bamboo lemur *Prolemur simus* and Black-and-white ruffed lemur *V. variagata*, as well as many other protected and threatened birds and mammals have been eaten by people in the study area. Game species such as the common tenrec *T. ecaudatus,* helmeted guinea fowl *Numida meleagris* and red-billed teal *Anas erythrorhyncha* are also widely reported as having been consumed.

There are three possible reasons why we find low levels of bushmeat consumption in the three-day recall whereas a high proportion of people report having ‘ever eaten’ bushmeat species. Firstly, if bushmeat represents a low proportion of the diet, most meals will not contain bushmeat. Secondly, asking people what they have eaten in the last three days is a sensitive question as people may feel that they incriminate themselves by admitting to eating protected or otherwise sensitive species [18]. Asking whether someone has ‘ever eaten’ a species is less incriminating which may mean informants feel less need to underplay the range of species they have consumed in their lifetime. Thirdly, diets and food availability may have changed and species consumed in the past may no longer consumed. Our field observations from over 10 years working in the area support our belief that the recall information is a relatively accurate reflection of diets. Protected species do not appear frequently in this data set as the vast majority of meals consist of no animal protein and when animal protein is consumed it mostly from domestic species, or wild non-protected species (mostly fish, aquatic invertebrates such as crabs and crayfish, or wild pig).

However, even occasional consumption of bushmeat by individual households can result in an important pressure on a species when human populations are high relative to area of natural habitat [39], or where targeted species have demographic characteristics making them vulnerable [40], [41]. The number of lemurs recorded by local monitors represents the minimum quantity that was killed in a sub-sample of our study villages and provides strong evidence that protected and threatened species are indeed killed regularly in at least one commune in the study area.

### Patterns of bushmeat consumption

Studies of diet among poor communities around the world have shown that wealth is an important predictor of consumption of animal protein [12]. Our three-day recall study confirmed that a higher proportion of meals consumed by urban, and wealthier, households contained meat. Wild meat consumption showed a similar, but less pronounced pattern (i.e. there was a less strong effect of being urban or of wealth on the consumption of wild meat). A study of the relationship between bushmeat consumption and income among Amerindian societies suggested an inverted U-shaped relationship; consumption increased with income at low income levels, but at higher income levels bushmeat consumption decreased with increasing income as people were able to afford expensive substitutes [42]. The fact that the relationship between consumption and wealth is weaker in our study for bushmeat than it is for meals containing any animal protein may be because people in higher wealth categories buy domestic meats where available. Because of the infrequency with which wild meat is consumed, we could not identify predictors of consumption of protected species, those of most concern to conservationists, from recall of meals eaten over the previous few days.

The data on whether a person has ‘ever eaten’ a given species reveals important differences between urban and rural populations. However predictors are not consistent across species; the brown lemur is more commonly eaten by poorer, rural people whereas the Madagascar flying fox is more widely eaten by richer, urban people. This reflects the fact that, in contrast to many African and south American countries [42], [43], Madagascar lacks an established commercial trade in all but a few bushmeat species. While bats and tenrecs often appear on restaurant menus and in markets in urban areas in western Madagascar [26], most urban people do not have access to species such as lemurs for which there is a very limited trade [15]. The differences in the trade are likely to be due at least in part to the legal status of species. Killing lemurs has been illegal since 1960 and this law is relatively well known making it difficult to sell lemurs openly. Fruit bats and some tenrecs are legally classified as game species so, although there are restrictions in terms of when they can be hunted [28], some sale is legal and anyone selling these species is likely to have less to fear from enforcement agencies. Other factors are also likely to influence the existence of an urban market in a particular species. Jones et al. [15] suggest that the lack of a large urban market for primate meat in Madagascar, in contrast to other African countries [2] may be at least in part due to traditional taboos against the consumption of large lemurs held by many ethnic groups.

In some parts of the world preferences for bushmeat over domestic meat, particularly by elite urban consumers, may play an important role in stimulating demand for rare species [13], [44] while the most preferred species in our study region are all domestic meats and fish. However this lack of preference for bushmeat may not be particularly unusual. Urban consumers in Equatorial Guinea distinguish less between bushmeat and domestic meat than between fresh and frozen [8], and rural people in Gabon are highly price sensitive with respect to their choice of meat, with taste playing a smaller role [9].

Of the protected species, the brown lemur was most preferred. Interestingly, this species is seldom listed by respondents as taboo, which may reflect its less human-like face and stance than other large diurnal lemurs. In the data set as a whole there is a clear relationship between taste preference and taboos; with species commonly listed as taboo getting very low rankings in terms of taste preference. It is well recognised that taboos can become internalized, affecting a person's perceptions. For example, secular Jews are often unable to enjoy foods considered forbidden by religious Jews [45]. Preference of course is only one driver of consumption among many. For example the seed-eating Vasa parrot is preferred by less than 1% of people but is commonly eaten. This is probably because it is killed as crop pests and then eaten rather than being targeted for food.

The role that traditional Malagasy taboos (*fady*) play in controlling hunting of certain protected and threatened species has been previously discussed [46], [47]. Such taboos have been credited with suppressing the demand for large diurnal lemurs, carnivores and hedgehog tenrecs [15], [48]. Relatively high proportions of people reported these species as taboo, lending support to the previous studies. However the degree to which such taboos offer long term protection is called into question by the high numbers of large diurnal lemurs being killed according to our local monitoring data. Whilst the high number of diademed sifaka recorded as bushmeat by local monitors is at least partly consistent with the results of our interviews, the large numbers of Indri observed are at odds with the low proportion of the population that admit to having eaten them and the high proportion claiming the species is taboo. We suggest that this may be because the area is undergoing rapid social change; affecting the power of traditional taboos to control hunting.

Rapid immigration is known to cause social change and is often associated with rapid economic development, such as mineral extraction or tourism [49], and immigrants to an area are less likely to respect local traditions. Mutschler et al. [50] attributed increase in hunting pressure on the Critically Endangered Lac Alaotra bamboo lemur to a decline in respect for, and adherence to, taboos preventing hunting. In the commune where our local monitoring was based, illegal artisanal gold mining began in 2007 and has become progressively more intense since. Our observations and conversations with local informants suggest that young men have more available cash and leisure time due to the transition from subsistence farming to panning for gold, and they spend more time in local bars, eating fried meat snacks with their drinks. Lemur hunting appears to have increased to supply this new market. It is also possible that people [especially young adults] who observe someone else consuming Indri without incurring any visible negative impacts may be more inclined to ignore the fady in the future. We suggest that the power of the taboo is declining, under twin pressures of increasing wealth and human mobility. This is not without precedent. Hunting for Indri has been reported from northern Madagascar and attributed to an influx of immigrants [51].

### Implications for conserving Malagasy wildlife

The depth and breadth of this study gives us confidence in our conclusions that the consumption of wild species, including those protected by law and threatened with extinction is prevalent in eastern Madagascar. This adds concrete evidence to the picture that is building up from scattered studies across the country that hunting is an important pressure on the country's native fauna [23], [26], [27].

Wildlife legislation in Madagascar was updated in 2006 and now provides legal protection to most threatened species as well as a framework for managing exploitation of game species [28]. Unfortunately wildlife laws are not well understood by local people [52] and communication of the existing laws would be an important first step to improved compliance. The deterrent effect of laws depends both on the size of the punishment and on the probability of being sanctioned [17], [53]. Both theory and empirical work has shown that very heavy sanctions are not always the most effective at reducing crime [54], for example, if the sanctions seem disproportionate to the crime, enforcement agents may be unwilling to press charges [17]. The legal sanctions for killing protected species in Madagascar are tough but there is flexibility in the fines and/or prison sentences. For example, for killing a lemur a person could expect a fine of between approximately US$5 and US$200 and/or between one month and two years in prison (Ordonnance 60–125). However our understanding is that these sanctions are seldom implemented. The limited resources available to enforcement agencies, and possibly a lack of will to prosecute wildlife crimes among the judiciary, needs to be addressed if illegal hunting is to be reduced.

It seems unlikely that the very large numbers of Indri and sifaka being killed in some of the villages reported by the local monitors could be sustained for long due to Indri's life history characteristics [27]. Informal interviews and observations do indeed suggest that heavy hunting in the area is a recent phenomenon which has developed as a result of social change in the area following the increase in gold mining. Indri are traditionally taboo in this part of Madagascar and hunting pressure was presumably lower in the past than for other, non-taboo lemur species [15], [48]. Evidence from our local monitors suggests that a large number of the Indri were killed by a few individuals who own guns and kill lemurs to sell. Relatively few individuals of other lemur species were recorded by the monitors. This may be either because hunting by subsistence hunters (who use traps which are particularly effective for brown lemurs)] was less well represented in the dataset. Or it may be because these other lemurs, not protected by taboos, have been hunted for longer in the area and so are less abundant and therefore make up a smaller proportion of the current harvest. Firearms are costly to use and in many rural societies are associated with elevated wealth (e.g. 10). In Madagascar, the legal use of guns and bullets requires permission from the Ministry of the Interior at the district level. Many of the firearms and bullets being used to hunt lemurs are fabricated locally and both these and conventional guns are unlikely to be legally owned [55]. Enforcing gun ownership laws in rural areas might be an effective way of reducing pressure on lemur species that are targeted by armed hunters because our impression is that there are relatively few commercial lemur hunters. Focusing enforcement in areas undergoing rapid social change may be particularly valuable where traditional institutions which may have offered protection in the past are breaking down.

Where bushmeat species are not preferred foods but are seen by consumers as substitutes for domestic meat or fish, the price of these alternatives will have a major impact on demand for bushmeat [42], [56]. In our study we found that consumers view bushmeat species as less preferred than domestic meat or fish, suggesting that the level of bushmeat consumption will be driven to a large extent by the price of these substitutes. Increasing the availability of domestic meat and fish through livestock rearing, small animal husbandry and aquaculture could therefore reduce the consumption of bushmeat [8]. However although this may reduce commercial hunting, it may have little effect on hunting for subsistence use.

Many of the species consumed such as the common tenrec, the Madagascar flying-fox and wildfowl such as the red-billed teal, are classed as game species. The government seeks to manage, rather than prevent, hunting of these species. Unfortunately, anecdotal evidence suggests that some game species are being over exploited [57]–[59], and one species, the Malagasy flying fox, was recently classified as Vulnerable by IUCN [60]. This is a problem both for the biodiversity of the country and for local people who use the meat. Very little is known about the biology of most of Madagascar's game species making sustainable management extremely challenging.

### Human welfare implications of controlling bushmeat hunting

A number of studies in Madagascar have mentioned the importance of bushmeat to local livelihoods and diets, particularly during periods of seasonal food shortage [24], [27], [61]. Rural Malagasy diets are very short of protein so if people who rely on bushmeat for at least part of their food are no longer able to hunt then some, already under nourished people, will suffer increased deprivation. However, efforts to reduce illegal hunting are necessary to protect the ecotourism industry upon which the livelihood of many people, including some rural poor, depends (directly or indirectly). To minimise the welfare implications of reducing illegal bushmeat hunting we suggest that illegal commercial hunting should be the primary target of enforcement measures and that there is increased effort to improve the availability of domestic animal protein (through improved availability of information on husbandry techniques and investment in veterinary extension work in rural areas).

Not all bushmeat hunting in Madagascar should be viewed as a conservation problem. Some species can be legally hunted and hunting of some species may be sustainable if managed properly. Parallel efforts to enable people to continue to hunt sustainably managed game species for subsistence purposes are therefore needed. There is evidence that in some cases traditional rules which managed harvested species (for example preventing hunting of pregnant tenrecs, or trapping of fruit bats at the roost) are breaking down over time (RKBJ and FHR unpublished data). Malagasy law allows for local laws (*dina*) to be entered formally into the legal system. There are a number of *dina* in existence in Madagascar which support these traditional management systems, however these need improved support from the regional authorities to be successful.

Beyond some preliminary studies demonstrating that Malagasy fruit bats may harbour dangerous viruses in the *Paramyxoviridae* family [62], almost nothing is known about the risk of disease transmission from wild species to humans via hunting in Madagascar. This is an area which clearly needs more research as the potential of zoonotic disease transmission through bushmeat hunting has not been considered by those formulating hunting policy in Madagascar.

### Conclusions

There is a growing body of evidence showing that wild animals in Madagascar are subject to locally high hunting pressure and that, whilst the bushmeat provide valuable protein, illegal hunting of protected species is becoming a major conservation issue. Recent publicity in Madagascar associated with seizures of lemur and tortoise bushmeat, or arrests of people involved in the bushmeat trade, has brought unprecedented attention to this issue [25], [63]. Since humans arrived in Madagascar, many of the island's largest terrestrial vertebrates have gone extinct, a loss blamed at least in part on hunting [64], [65]. If further extinctions are to be avoided, urgent action is needed to reduce hunting of protected species. The progress in setting up new protected areas in Madagascar, which is adding large areas of forest and wetland to the national reserve system, needs to be accompanied by an urgent initiative to address hunting.

## Supporting Information

Figure S1 **Map of the study area.**
(TIF)

Table S1 **Species included in the interviews including their threat status according to IUCN and their legal status under Malagasy law.**
(DOCX)

Table S2 **Summary of correlations between the predictor variables considered for modelling.**
(DOCX)

Table S3 **Summary of model selection for three day recall models.**
(DOCX)

Table S4 **Estimated coefficients for best-fitting three day recall model.**
(DOCX)

Table S5 **Summary of model selection for lifetime consumption models.**
(DOCX)

Table S6 **Estimated coefficients for best-fitting lifetime consumption model.**
(DOCX)

Table S7 **Summary of the estimated number of lemurs killed in nine locations (from local monitor data).**
(DOCX)
