# Supplementary material for: Analysis of Patterns of Bushmeat Consumption Reveals Extensive Exploitation of Protected Species in Eastern Madagascar
Source: PLoS One. 2011 Dec 14;6(12):e27570. doi: 10.1371/journal.pone.0027570 (PMC3237412; doi:10.1371/journal.pone.0027570)
Supplement: Table S1 — Species included in the interviews including their threat status according to IUCN and their legal status under Malagasy law. (DOCX) [file pone.0027570.s002.docx]

*Table S1: Species included in the interviews including their threat status according to IUCN and their legal status under Malagasy law.*

| *Common name* | *Scientific name* | *Legal Status* | *Red List Status* |
| --- | --- | --- | --- |
| Black-and-white ruffed lemur | *Varecia variegata* | Strictly protected | CR |
| Broad-striped Malagasy mongoose | *Galidictis fasciata* | Strictly protected | NT |
| Brown lemur | *Eulemur fulvus* | Strictly protected | NT |
| Diademed sifaka | *Propithecus diadema* | Strictly protected | EN |
| Dwarf lemur spp. | *Cheirogaleus spp.* | Strictly protected | Various |
| Gmelin’s woolly lemur | *Avahi laniger* | Strictly protected | LC |
| Greater bamboo lemur | *Prolemur simus* | Strictly protected | CR |
| Indri | *Indri indri* | Strictly protected | EN |
| Eastern Lesser Bamboo Lemur | *Hapalemur griseus* | Strictly protected | VU |
| Madagascar crested ibis | *Lophotibis cristata* | Strictly protected | NT |
| Meller's duck | *Anas melleri* | Strictly protected | EN |
| Mouse lemur spp. | *Microcebus spp.* | Strictly protected | Various |
| Red-bellied lemur | *Eulemur rubriventer* | Strictly protected | VU |
| Ring-tailed mongoose | *Galidia elegans* | Strictly protected | LC |
| Crested coua | *Coua cristata* | Protected | LC |
| Falanouc | *Eupleres goudoti* | Protected | NT |
| Fossa | *Cryptoprocta ferox* | Protected | VU |
| Hedgehog tenrec | *Setifer setosus* | Protected | LC |
| Lowland streaked-tenrec | *Hemicentetes semispinosus* | Protected | LC |
| Malagsy striped civet | *Fossa fossana* | Protected | NT |
| Sportive lemur spp. | *Lepilemur spp.* | Protected | Various |
| Vasa parrot spp. | *Coracopsis spp.* | Protected | LC |
| Common tenrec | *Tenrec ecaudatus* | Game | LC |
| Helmeted guinea fowl | *Numida meleagris* | Game | LC |
| Knob-billed duck | *Sarkidiornis melanotos* | Game | LC |
| Madagascar flying fox | *Pteropus rufus* | Game | VU |
| Madagascar rousette | *Rousettus madagascariensis* | Game | NT |
| Madagascar straw-coloured fruit bat | *Eidolon dupreanum* | Game | VU |
| Red-billed teal | *Anas erythrorhyncha* | Game | LC |
| White-faced whistling duck | *Dendrocygna viduata* | Game | LC |
| Bush pig | *Potamochoerus larvatus* | Nuisance | LC |
| Chicken (domestic) | *Gallus gallus* | Domestic | Not listed |
| Goat (domestic) | *Capra spp.* | Domestic | Not listed |
| Goose (domestic) | *Anser spp.* | Domestic | Not listed |
| Turkey (domestic) | *Meleagris gallapavo* | Domestic | Not evaluated |
| Zebu (domestic) | *Bos taurus* | Domestic | Not evaluated |
| Boa spp. | *Acrantophis sp.* | Not listed | VU |
| Chonophorus macrorhynchus | *Chonophorus macrorhynchus* | Not listed | Not evaluated |
| Eel spp. | *Anguilla spp.* | Not listed | Not listed |
| Freshwater crab spp. | *Hydrothelphusa spp.* | Not listed | Various |
| Freshwater crayfish spp. | *Astacoides spp.* | Not listed | Various |
| Freshwater shrimp | *Macrobrachium spp.* | Not listed | Various |
| Freshwater turtle | *Pelusios subniger* | Not listed | LC |
| Indian civet | *Viverricula indica* | Not listed | LC |
| Pig (domestic) | *Sus scrofa* | Not listed | Not evaluated |
| Rabbit (domestic) | *Ortyctolagus spp.* | Not listed | Not evaluated |
| Rainbow fish | -- | Not listed | Not evaluated |
| Tilapia spp. | *Tilapia spp.* | Not listed | Not evaluated |
| Mantidactylus frog spp. | *Mantidactylus spp.* | Various | Various |
| Microbat spp. | *Microchiroptera spp.* | Various | Various |
