# Supplementary material for: Analysis of Patterns of Bushmeat Consumption Reveals Extensive Exploitation of Protected Species in Eastern Madagascar
Source: PLoS One. 2011 Dec 14;6(12):e27570. doi: 10.1371/journal.pone.0027570 (PMC3237412; doi:10.1371/journal.pone.0027570)
Supplement: Table S2 — Summary of correlations between the predictor variables considered for modelling. (DOCX) [file pone.0027570.s003.docx]

|  | *urban* | *resident* | *livelihood* | *fuel type* | *no. rooms* | *no. occupants* | *season* |
| --- | --- | --- | --- | --- | --- | --- | --- |
| *urban* | 1.00 | 0.06 | 0.61 | 0.64 | 0.19 | 0.07 | 0.70 |
| *resident* |  | 1.00 | 0.27 | 0.16 | 0.10 | 0.05 | 0.04 |
| *livelihood* |  |  | 1.00 | 0.58 | 0.24 | 0.04 | 0.45 |
| *fuel type* |  |  |  | 1.00 | 0.26 | 0.05 | 0.40 |
| *no. rooms* |  |  |  |  | 1.00 | 0.23 | 0.11 |
| *no. occupants* |  |  |  |  |  | 1.00 | 0.10 |
| *season* |  |  |  |  |  |  | 1.00 |
