# Supplementary material for: Analysis of Patterns of Bushmeat Consumption Reveals Extensive Exploitation of Protected Species in Eastern Madagascar
Source: PLoS One. 2011 Dec 14;6(12):e27570. doi: 10.1371/journal.pone.0027570 (PMC3237412; doi:10.1371/journal.pone.0027570)
Supplement: Table S3 — Summary of model selection for three day recall models. (DOCX) [file pone.0027570.s004.docx]

| *model* | *AIC* | *delta AIC* | *w* |
| --- | --- | --- | --- |
| urban + resident + no. rooms | 4810.88 | 0 | 1.00 |
| urban + no. rooms | 4873.46 | 62.57 | 0.00 |
| urban + resident | 4875.39 | 64.51 | 0.00 |
| resident + no. rooms | 4931.69 | 120.80 | 0.00 |
| urban | 4947.42 | 136.53 | 0.00 |
| no. rooms | 4998.41 | 187.52 | 0.00 |
| resident | 5011.57 | 200.68 | 0.00 |
