# Supplementary material for: Analysis of Patterns of Bushmeat Consumption Reveals Extensive Exploitation of Protected Species in Eastern Madagascar
Source: PLoS One. 2011 Dec 14;6(12):e27570. doi: 10.1371/journal.pone.0027570 (PMC3237412; doi:10.1371/journal.pone.0027570)
Supplement: Table S4 — Estimated coefficients for best-fitting three day recall model. (DOCX) [file pone.0027570.s005.docx]

|  | *Domestic meat* | *Wild meat* |
| --- | --- | --- |
| Intercept | -2.30 (SE = 0.15) | -1.72 (SE = 0.12) |
| Urban | 1.50 (SE = 0.14) | 1.05 (SE = 0.14) |
| Resident | -1.87 (SE = 0.11) | -0.50 (SE = 0.11) |
| No. rooms (2) | 0.91 (SE = 0.15) | 0.29 (SE = 0.12) |
| No. rooms (3) | 1.31 (SE = 0.17) | 0.50 (SE = 0.15) |
