# Supplementary material for: Analysis of Patterns of Bushmeat Consumption Reveals Extensive Exploitation of Protected Species in Eastern Madagascar
Source: PLoS One. 2011 Dec 14;6(12):e27570. doi: 10.1371/journal.pone.0027570 (PMC3237412; doi:10.1371/journal.pone.0027570)
Supplement: Table S5 — Summary of model selection for lifetime consumption models. (DOCX) [file pone.0027570.s006.docx]

| *model* | *AIC* | *delta AIC* | *w* |
| --- | --- | --- | --- |
| species + urban + resident + no.rooms + urban:species + resident:species + no.rooms:species | 32160.91 | 0.00 | 1.00 |
| species + urban + resident + no.rooms + urban:species + resident:species | 32251.96 | 91.05 | 0.00 |
| species + urban + resident + urban:species + resident:species | 32271.53 | 110.61 | 0.00 |
| species + urban + resident + no.rooms + urban:species + no.rooms:species | 32509.74 | 348.83 | 0.00 |
| species + urban + no.rooms + urban:species + no.rooms:species | 32513.31 | 352.39 | 0.00 |
| species + urban + resident + no.rooms + resident:species + no.rooms:species | 32520.33 | 359.42 | 0.00 |
| species + resident + no.rooms + resident:species + no.rooms:species | 32692.35 | 531.43 | 0.00 |
