# Supplementary material for: Analysis of Patterns of Bushmeat Consumption Reveals Extensive Exploitation of Protected Species in Eastern Madagascar
Source: PLoS One. 2011 Dec 14;6(12):e27570. doi: 10.1371/journal.pone.0027570 (PMC3237412; doi:10.1371/journal.pone.0027570)
Supplement: Table S6 — Estimated coefficients for best-fitting lifetime consumption model. (DOCX) [file pone.0027570.s007.docx]

| **Main effects** | *Intercept* | *Urban* | *Resident* | *Rooms (2)* | *Rooms(3+)* |
| --- | --- | --- | --- | --- | --- |
|  |  |  |  |  |  |
|  | **0.93 (SE = 0.22)***** | **-1.03 (SE = 0.26)***** | **-0.41 (SE = 0.20)*** | **0.89 (SE = 0.21)***** | -0.01 (SE = 0.26) |
|  |  |  |  |  |  |
| **Species specific effects & species interactions** | *Species effect* | *Species:Urban* | *Species:Resident* | *Species:Rooms (2)* | *Species:Rooms(3+)* |
| *Anas melleri* | **-0.63 (SE = 0.29)*** | **-1.66 (SE = 0.39)***** | 0.08 (SE = 0.26) | **-0.71 (SE = 0.27)**** | -0.12 (SE = 0.34) |
| *Avahi laniger* | **-1.03 (SE = 0.26)***** | **-2.33 (SE = 0.46)***** | **0.72 (SE = 0.24)**** | **-0.79 (SE = 0.25)**** | -0.57 (SE = 0.31) |
| *Cheirogaleus spp.* | **-0.69 (SE = 0.26)**** | **-1.82 (SE = 0.39)***** | 0.35 (SE = 0.24) | **-0.67 (SE = 0.25)**** | -0.23 (SE = 0.31) |
| *Coracopsis spp.* | 0.040 (SE = 0.28) | **-2.25 (SE = 0.36)***** | **1.004795 (SE = 0.25)***** | **-0.64 (SE = 0.28)*** | -0.28 (SE = 0.33) |
| *Coua cristata* | **-1.25 (SE = 0.27)***** | **-2.16 (SE = 0.47)***** | **0.47 (SE = 0.24)*** | **-0.99 (SE = 0.25)***** | -0.15 (SE = 0.31) |
| *Cryptoprocta ferox* | **-2.49 (SE = 0.28)***** | -0.85 (SE = 0.44) | **0.58 (SE = 0.25)*** | **-0.66 (SE = 0.26)*** | -0.07 (SE = 0.33) |
| *Dendrocygna viduata* | **-2.11 (SE = 0.27)***** | -0.10 (SE = 0.36) | 0.13 (SE = 0.24) | **-0.51 (SE = 0.26)*** | **0.67 (SE = 0.31)*** |
| *Eidolon dupreanum* | **-2.10 (SE = 0.29)***** | **-1.93 (SE = 0.64)**** | **-1.18 (SE = 0.26)***** | 0.02 (SE = 0.29) | -0.05 (SE = 0.36) |
| *Eulemur fulvus* | -0.40 (SE = 0.26) | **-1.82 (SE = 0.37)***** | **0.59 (SE = 0.24)*** | **-0.70 (SE = 0.25)**** | -0.50 (SE = 0.31) |
| *Eulemur rubriventer* | -0.49 (SE = 0.27) | **-2.38 (SE = 0.46)***** | -0.12 (SE = 0.24) | **-0.68 (SE = 0.25)**** | **-0.83 (SE = 0.31)**** |
| *Eupleres goudoti* | **-1.75 (SE = 0.28)***** | **-1.59 (SE = 0.51)**** | -0.35 (SE = 0.25) | -0.34 (SE = 0.26) | -0.27 (SE = 0.34) |
| *Fossa fossana* | **-1.18 (SE = 0.27)***** | **-1.37 (SE = 0.43)**** | -0.08 (SE = 0.25) | **-0.63 (SE = 0.26)*** | **-0.69 (SE = 0.33)*** |
| *Galidia elegans* | **-1.65 (SE = 0.27)***** | **-2.14 (SE = 0.57)***** | -0.32 (SE = 0.24) | -0.30 (SE = 0.26) | -0.46 (SE = 0.33) |
| *Galidictis fasciata* | **-2.09 (SE = 0.29)***** | **-2.56 (SE = 0.83)**** | **-0.66 (SE = 0.26)*** | -0.33 (SE = 0.28) | **-0.93 (SE = 0.40)*** |
| *Hapalemur griseus* | **-0.65 (SE = 0.26)*** | **-1.68 (SE = 0.38)***** | **0.63 (SE = 0.24)**** | **-0.95 (SE = 0.25)***** | **-0.74 (SE = 0.31)*** |
| *Hemicentetes semispinosus* | **1.31 (SE = 0.31)***** | -0.20 (SE = 0.34) | 0.53 (SE = 0.28) | -0.41 (SE = 0.29) | -0.04 (SE = 0.35) |
| *Indri indri* | **-3.55 (SE = 0.31)***** | 0.19 (SE = 0.45) | **0.88 (SE = 0.28)**** | **-0.90 (SE = 0.29)**** | -0.32 (SE = 0.37) |
| *Lepilemur spp.* | **-1.01 (SE = 0.27)***** | **-2.65 (SE = 0.52)***** | **0.68 (SE = 0.24)**** | **-1.06 (SE = 0.25)***** | **-0.85 (SE = 0.32)**** |
| *Leptosomus discolor* | **-4.24 (SE = 0.43)***** | -0.51 (SE = 0.82) | 0.30 (SE = 0.38) | **-0.85 (SE = 0.40)*** | -1.07 (SE = 0.66) |
| *Lophotibis cristata* | -0.25 (SE = 0.29) | **-2.83 (SE = 0.48)***** | 0.28 (SE = 0.26) | **-0.87 (SE = 0.28)**** | **-1.19 (SE = 0.35)***** |
| *Microcebus spp.* | -0.16 (SE = 0.27) | **-2.58 (SE = 0.43)***** | -0.34 (SE = 0.24) | -0.40 (SE = 0.25) | 0.10 (SE = 0.31) |
| *Numida meleagris* | **-1.15 (SE = 0.26)***** | **0.75 (SE = 0.32)*** | 0.44 (SE = 0.23) | **-0.62 (SE = 0.25)*** | **0.60 (SE = 0.30)*** |
| *Pelomedusa subrufa* | **-1.24 (SE = 0.27)***** | **0.98 (SE = 0.32)**** | 0.29 (SE = 0.24) | **-0.90 (SE = 0.26)***** | 0.47 (SE = 0.31) |
| *Prolemur simus* | **-1.61 (SE = 0.29)***** | **-3.83 (SE = 1.12)***** | **-0.95 (SE = 0.26)***** | -0.27 (SE = 0.29) | -0.69 (SE = 0.38) |
| *Propithecus diadema* | **-0.93 (SE = 0.26)***** | **-1.50 (SE = 0.38)***** | **0.72 (SE = 0.24)**** | **-0.76 (SE = 0.25)**** | **-0.85 (SE = 0.31)**** |
| *Pteropus rufus* | **-1.47 (SE = 0.26)***** | **1.34 (SE = 0.32)***** | -0.45 (SE = 0.23) | -0.02 (SE = 0.25) | **0.73 (SE = 0.30)*** |
| *Rousettus madagascariensis* | **-2.22 (SE = 0.31)***** | **-2.11 (SE = 0.71)**** | **-1.21 (SE = 0.27)***** | -0.06 (SE = 0.31) | -0.07 (SE = 0.39) |
| *Sarkidiornis melanotos* | **-3.11 (SE = 0.29)***** | **-1.02 (SE = 0.50)*** | **1.96 (SE = 0.27)***** | **-1.13 (SE = 0.26)***** | -0.42 (SE = 0.33) |
| *Setifer setosus* | -0.12 (SE = 0.27) | 0.46 (SE = 0.32) | 0.03 (SE = 0.25) | -0.46 (SE = 0.25) | 0.27 (SE = 0.31) |
| *Tenrec ecaudatus* | **2.38 (SE = 0.34)***** | **-1.06 (SE = 0.34)**** | -0.20 (SE = 0.30) | -0.48 (SE = 0.31) | -0.15 (SE = 0.36) |
| *Varecia variegata* | **-1.84 (SE = 0.30)***** | **-1.16 (SE = 0.49)*** | **-0.63 (SE = 0.27)*** | -0.35 (SE = 0.29) | -0.58 (SE = 0.39) |
