# Supplementary material for: Analysis of Patterns of Bushmeat Consumption Reveals Extensive Exploitation of Protected Species in Eastern Madagascar
Source: PLoS One. 2011 Dec 14;6(12):e27570. doi: 10.1371/journal.pone.0027570 (PMC3237412; doi:10.1371/journal.pone.0027570)
Supplement: Table S7 — Summary of the estimated number of lemurs killed in nine locations (from local monitor data). (DOCX) [file pone.0027570.s008.docx]

|  | Village 1 (21 months) | Town 1 (18 months) | Town 1 (16 months) | Town 1 (10 months) | Village 2 (12 months) | Village 3 (12 months) | Village 4 (12 months) | Village 5 (5 months) | Village 5 (4 months) | Village 5 (6 months) | Village 6 (7 months) | Village 7 (6 months) | Village 8 (6 months) | **Total (135)** |
| --- | --- | --- | --- | --- | --- | --- | --- | --- | --- | --- | --- | --- | --- | --- |
| *Propithecus* | 11 | 18 | 17 | 6 | 5 | 0 | 1 | 8 | 0 | 4 | 28 | 4 | 19 | **121** |
| *Indri* | 96 | 42 | 21 | 20 | 3 | 0 | 18 | 4 | 6 | 5 | 8 | 4 | 6 | **233** |
| *Hapalemur* | 0 | 0 | 0 | 0 | 1 | 0 | 1 | 0 | 0 | 1 | 1 | 0 | 0 | **4** |
| *Avahi* | 7 | 17 | 0 | 4 | 1 | 1 | 1 | 0 | 0 | 0 | 2 | 0 | 0 | **33** |
| *Cheirogaleus* | 5 | 1 | 0 | 0 | 3 | 0 | 0 | 0 | 0 | 0 | 0 | 0 | 9 | **18** |
| *Microcebus* | 0 | 0 | 0 | 0 | 1 | 0 | 0 | 0 | 0 | 0 | 0 | 0 | 6 | **7** |
| *Eulemur* | 3 | 6 | 1 | 2 | 5 | 0 | 1 | 2 | 0 | 2 | 2 | 0 | 24 | **48** |
| *Varecia* | 5 | 0 | 2 | 0 | 0 | 0 | 0 | 0 | 0 | 0 | 0 | 0 | 2 | **9** |
| *Lepilemur* | 0 | 0 | 0 | 0 | 0 | 0 | 0 | 0 | 0 | 0 | 0 | 4 | 6 | **10** |
|  | **127** | **84** | **41** | **32** | **19** | **1** | **22** | **14** | **6** | **12** | **41** | **12** | **72** | **483** |
